# On the Cause of Intermittent Fever

**Published:** 1866-03

**Authors:** Henry M. Lyman

**Affiliations:** Pathologist to Cook County Hospital


### CHICAGO MEDICAL JOURNAL.

VOL. XXIII. MARCH, 1866. No. 3.

### ORIGINAL CONTRIBUTIONS.

*On the Cause of Intermittent Fever.* By HENRY M. LYMAN, M. D., Pathologist to Cook County Hospital.

The January number of the *American Journal of the Medical Sciences* contains an article from the pen of Prof. J. H. Salisbury, of Cleveland, announcing the discovery of the long-sought cause of malarial diseases. The Professor has already distinguished himself as a skilful microscopist and physiologist. The readers of the *American Medical Times* will readily recall to mind a paper on a form of measles originating from a species of fungus produced on damp straw, in which communication Dr. S. suggested a vegetable origin for the camp measles, which was at that time prevailing so extensively in the U. S. Army. Continuing his observations with the microscope, and extending their field, he has at length satisfied himself that the old hypothesis of a cryptogamic origin of the miasmatic poison is correct. The announcement of this discovery constitutes one of the most interesting papers we have ever read,—one which deserves the careful study of every physician throughout the world. If the statements of Prof. Salisbury are confirmed by equally skilled observers in other regions and other climates where miasmatic fevers prevail, his name will rank among the first of the great names of observers who have advanced the cause of science.

It is impossible to refrain from admiration of the patience and perseverance displayed by Dr. S. in the conduct of his researches. For three years, his time was devoted to microscopical investigation of the exhalations of the soil in miasmatic localities. His method was ingenious. Examining the expectoration and urine of patients suffering from miasmatic fever, he discovered in these excretions a variety of microscopical cells of vegetable origin. Only one form, however, was invariably present—a minute oblong cell, "consisting of a distinct nucleus, surrounded by a smooth cell-wall, with a highly clear, apparently empty, space between the outer cell-wall and nucleus." These little bodies were also recognized in the night air of miasmatic regions. To discover their source was the problem which Dr. S. proposed to himself.

Repeating the experiment of the Italian savans, he suspended plates of glass in the vicinity of swampy soil; but, unlike his predecessors in the inquiry, who had been content with the detection of an organic residuum in the dew that condensed upon the glass, he subjected the drops of water thus obtained to microscopical analysis, and finally discovered that the cells, which had been remarked in the expectoration of fever patients, were the spores of a cryptogamic plant which appeared upon freshly turned earth like "a whitish mould, or, more closely, the incrustation of some salt." By exposing plates of glass, dipped in a solution of chloride of calcium, he ascertained:

1. "That cryptogamic spores and other minute bodies are mainly elevated above the surface during the night. That they rise and are suspended in the cold, damp exhalation from the soil, after the sun has set, and that they fall again to the earth soon after the sun rises.

2. "That in the latitude of Ohio, these bodies seldom rise above from thirty-five to sixty feet above the low levels. That in the northern and central portions of the State, they rise from thirty-five to forty-five feet, while in the southern, from forty to sixty feet.

3. "That at Nashville and Memphis they rise from sixty to one hundred feet and more above the surface.

4. "That above the summit plane of the cool night exhalations, these bodies do not rise, and intermittents do not extend.

5. "That the day air of malarial districts is quite free from these palmelloid spores, and from causes that produce intermittents."

Having satisfied himself that the "palmelloid spores" were the cause of malarial fever, Dr. S. proceeded to examine the night air of every locality where ague prevailed, and was successful in discovering the microscopic agent in every instance. The often-observed phenomena of the occurrence of intermittent fever in the track of winds blowing over marshy regions, he at once stripped of mystery by displaying under his object-glass the poisonous spores whose flight he had intercepted. Fevers which broke out in localities where the disease had never before been experienced were thus traced to adjacent excavations of the soil, and were arrested through the destruction of their cause by covering the cryptogamic crop with straw or with caustic lime. The Professor at length put his theory to the test of the following experiment:

"I filled six tin boxes with the surface earth from a decidedly malarious drying prairie bog, which was covered completely with the palmellæ previously described. Cakes of the surface soil were cut out, the size and depth of the boxes, and carefully fitted in without disturbing more than possible the surface vegetation. The covers were then placed on, and the boxes transported to a high, hilly district, some five miles distant from any malarious locality, and where a case of ague had never been known to occur. The locality was over three hundred feet above the stream levels, was dry, sandy and rocky. I here placed the boxes of cryptogams on the sill of an open second-story window, opening into the sleeping apartment of two young men; removed the covers, and gave particular directions, that the boxes should not be disturbed, and the window left open. On suspending a plate of glass over the boxes on the fourth day, during the night, the under surface of the plate on the following morning was found covered with palmelloid spores, and numerous cells of the same kind adhered to a suspended plate in the room, which was moistened with a concentrated solution of chloride of calcium.

"On the twelfth day, one of the young men had a well-marked paroxysm of ague, and on the fourteenth, the other was taken down with the disease. They both began to feel unnatural and dull about the sixth day. All three stages of the paroxysms were well marked. The type in both cases was tertian, and was readily controlled by the appropriate remedies.

"Four members of the family slept on the lower floor of the house, but none of them were affected."

A few other experiments of the same nature were tried with similar results.

Prof. Salisbury's theory of the cause of intermittent fever, then, may be thus briefly stated:

Certain cryptogamic forms of vegetation are produced upon drying soils, usually where the earth has been recently disturbed by any cause.

These growths produce an almost infinite number of spores, which are dispersed by the damp air of the night, and which by contact with the mucous surfaces of the human body, excite "local fever," which results in either intermittent or remittent fever.

Had the Professor at this point laid down his pen, and written no more, we should have been disposed to put faith in his discovery. But he proceeds still further, and not only makes assertions but draws conclusions which lead us to fear that he has not fully proved his point. In one instance, at least, his data are lacking in accuracy. We refer to his account of the fevers which occurred on College Hill, at Nashville, during the late war. Dr. S. supposes that the fortifications which were thrown up on the hill, in the vicinity of the hospitals, occasioned the production of the severe congestive fevers, which in several instances proved fatal to inmates of the hospitals. Visiting the spot, he found that "the soil on the perpendicular sides of the ditch, which was dug to strengthen the place, was covered completely with cryptogamic vegetation * * * similar to those

(palmellæ) in districts where intermittents are of a congestive type."

Having been ourself on duty in the hospitals on College Hill, during the time of which he speaks, and having barely escaped death from congestive intermittent fever contracted on the spot, we can positively assure the Professor, that the disease could not have been traced to the fortifications in question, for the reason that they were not then in existence. It was the month of September, a considerable time after the occurrence of our worst cases of fever, before the hospitals were removed and the excavations were made which Dr. Salisbury explored. During our occupation of the hill, in the summer of 1862, the only instance in which the soil was disturbed was the excavation of a privy; but, according to the Professor's own theory, the effects of this must have been neutralized by the quicklime which was freely scattered in and around the pit. We discovered, after extended inquiry, that, though the physicians of Nashville would not admit the existence of malaria within the city or in its vicinity, there was in the minds of the unprofessional public a firm conviction that College Hill was a peculiarly miasmatic locality, and that it had always been unhealthy from the earliest settlement of the country.

A slight inaccuracy of this sort, however, is by no means fatal to the argument, for Dr. S. distinctly asserts that "occasionally the soil on the hill, where it had not been disturbed, was covered slightly with this same vegetation." Our incredulity has its origin in other sources. We are not prepared to deny that these "palmelloid spores" may be in some way the cause of intermittent fever, but we do not think that such has yet been proved to be the fact. After examining the urine of several hundred cases of intermittent and remittent fever, and finding cryptogamic spores always present in the urine as well as in the expectoration, Prof. S. considers it safe to say that the results of these examinations "establish the fact that ague plants, the same as grown upon the ague soil, are constantly developing in the system of the intermittent fever patient; and that the urinary organs constitute one important outlet for the elimination of this fever vegetation." From this it logically follows that, to effect a cure of the disease, "this exciting cause must be carried out of the organism through those excretory channels which nature has provided for the elimination of effete and abnormal products. * * * The sweating stage of the paroxysm of ague is essentially a curative one." The Professor believes then, that the cryptogamic spores find their way into the circulation, where they grow and multiply, poisoning the blood of the patient, and keeping up a series of abnormal manifestations, until they are finally expelled from the system. If this be true, here is a discovery greater than the discovery of the origin of the vegetation itself. Dr. Lionel S. Beale, one of the highest authorities in matters of this sort, declares that "it is very doubtful if the growth and multiplication of vegetable germs can proceed in the circulating fluids of a living body without causing death in a very short time; for the conditions favorable to their existence and multiplication are incompatible with the life of the germinal matter of the higher tissues. The germination and multiplication of very low vegetable or animal organisms in man and the higher animals, are an indication not only that the death of the tissue has taken place, but that it is passing into a state of decomposition. It is true, that bacteria have been detected in the blood of patients during life, but hitherto, only in cases shortly before death, at a time, when the blood in which they grew and multiplied was no longer fit for nourishing the tissues, and was itself passing into decomposition. Millions of bacteria exist in the softened outer portions of the fibrinous clot of an aneurismal sac, and therefore, in such close proximity to the blood that it is almost certain that, from time to time, some pass into the current of the circulation; but, if this were the case, they would be destroyed, or else so altered, that they would cease to multiply. For, before multiplication could take place, changes must have occurred in the composition of the blood which would render it incapable of supporting the life of its owner."

To infer therefore with Prof. Salisbury, that because "palmelloid spores" are found near both ends of the circulatory channel, they also pervade the system itself, seems to us illogical. These organisms, even if discovered in the pelvis of the kidney, cannot consequently be supposed to multiply and to act *within the patient*, in the strict sense of that phrase, any more than the Sarcina which sometimes grows in the stomach, or the Pennicellia, Aspergilli, or Spherotheci, which are also found in the urine of intermittent fever patients. These growths, the Professor recognises as consequences of diseased action, producing conditions favorable to cryptogamic life in the urinary excretion. If it be true, as he asserts, that "in these obstinate cases of the disease, the urine passes rapidly to the acetous fermentation even before it is voided," we can see no reason why the bladder should not be occupied by "palmelloid spores," which have been produced *in situ*. That they are the fruits of a crop which has been nurtured in the circulation, we cannot believe until some one makes a satisfactory demonstration of cryptogamic growth in the current of the blood. We have examined this essay in vain for some note of the appearances of the circulating fluids of patients suffering with intermittent fever. If these spores flourish in the blood and are thrown off with the urine, the Professor can inform us whether the perspiration also contains them; and if they are easy of recognition in the secretions and excretions, their detection should not be very difficult in the fluids of the body, provided, they do actually exist and multiply therein. And, if we believe with the Professor, that the phenomena of intermittent fever are the efforts of nature to eliminate from the system the poison which caused the disease,* will not failure to discover these cryptogamic organisms within the system of the patient prove that the poison to be eliminated is, after all, really something of a different nature?

Dr. Salisbury's paper forms a valuable contribution to our knowledge of the forms of cryptogamic vegetation which flourish in miasmatic localities. We hope that similar observations may be undertaken wherever malarial diseases exist. If it can be satisfactorily proved that these peculiar growths are uniformly present in malarious atmosphere, and that without them intermittent fever cannot be excited, we shall be disposed to believe that, if not the cause, they at least

* "When the tissues have become poisoned to a certain extent, there is a reaction on the part of the system, an effort of nature to eliminate the poisonous products already in the body. This effort is the paroxysm, which constitutes what we call the disease."

co-exist with the cause of the disease; and that they afford the most certain means of recognizing the presence of the miasmatic agent; or, if we may be allowed to drop the theory of blood poisoning, and to assign the phenomena of intermittent fever to the same category with the morbid appearances caused by contact with the exhalations of the poison ivy, it will not be difficult to accept the Doctor's theory of the cause as it stands. Whichever horn of the dilemma is accepted, *more light* is all we ask.

